# Editorial: Hallucinations: New Interventions Supporting People with Distressing Voices and/or Visions

**DOI:** 10.3389/fpsyg.2016.01418

**Published:** 2016-09-21

**Authors:** Simon McCarthy-Jones, Mark Hayward, Flavie Waters, Iris E. Sommer

**Affiliations:** ^1^Department of Psychiatry, Trinity College DublinDublin, Ireland; ^2^Sussex Partnership NHS Foundation Trust, University of SussexBrighton, UK; ^3^Clinical Research Centre, North Metro Health Service Mental HealthPerth, WA, Australia; ^4^School of Psychiatry and Clinical Neurosciences, University of Western AustraliaPerth, WA, Australia; ^5^Neuroscience Division, Rudolf Magnus Institute for Neuroscience, University Medical Center UtrechtUtrecht, Netherlands

**Keywords:** psychosis, schizophrenia, post-traumatic stress disorder, cognitive behavioral therapy, traumatology

Hallucinations can occur across the five sensory modalities (auditory, visual, olfactory, tactile, and gustatory). Whilst they have the potential to be benign or even highly valued (Romme et al., 2009; Sommer et al., 2010), they can often be devastating experiences associated with distress, impaired social, and occupational functioning, self-harm and suicide (McCarthy-Jones et al., 2013; Kjelby et al., 2015). Those who experience hallucinations in this latter manner may do so within the context of a wide range of psychiatric diagnoses, including schizophrenia, bipolar disorder, borderline personality disorder, and post-traumatic stress disorder (Blom and Sommer, 2011; Larøi et al., 2012; McCarthy-Jones, 2012). The only routinely available interventions for people distressed by hallucinations are antipsychotic drugs, which date from the introduction of chlorpromazine in the 1950s, and manualized cognitive behavioral therapy, which originated in the 1990s. These interventions do not help all people distressed by hallucinations (Lecrubier et al., 2007; Sommer et al., 2012; Jauhar et al., 2014; van der Gaag et al., 2014), and in the case of antipsychotic medication, come with notable side-effects. There has hence been great interest in new interventions to support people distressed by hallucinations.

The goal of this Frontiers Research Topic is to present a collection of papers on new developments in clinical interventions for those distressed by hallucinations. In the psychiatric condition that remains most strongly associated with hallucinations, schizophrenia, the majority (~70%) of people will have experienced hallucinations in the auditory modality (Thomas et al., 2007; McCarthy-Jones et al., Submitted), approximately a third will have experienced visual hallucinations (Waters et al., 2014; McCarthy-Jones et al., Submitted), and a smaller minority will have experienced hallucinations in other modalities (Thomas et al., 2007; McCarthy-Jones et al., Submitted). Consistent with this prevalence, this collection focusses on auditory and visual hallucinations. This is not to minimize the potential distress that can occur from hallucinations in other modalities. For example, tactile hallucinations, particularly when stemming from earlier experiences of sexual abuse (Read et al., 2003), can be highly distressing, and improved ways to help sufferers of such experiences are also needed.

This collection starts with a paper by Coebergh et al. offering insights into how musical hallucinations may be treated. An interesting theoretical counterpoint to this paper is offered by Deamer and Wilkinson, who stress how auditory verbal hallucinations (AVH; “hearing voices”) may be understood primarily as hallucinated acts of communication, rather than hallucinated sounds, and then consider how this conception of AVH may help us better understand some recent therapeutic successes.

Two papers in this collection focus on the therapeutic implications of the now burgeoning research literature showing traumatic life events are associated with hallucinations (e.g., Read and Argyle, 1999; Read et al., 2003; Janssen et al., 2004; Bentall et al., 2012). Whilst not all individuals who experience hallucinations will have experienced traumatic life events, this is increasingly emerging as a notable causal factor (Janssen et al., 2004; Bentall et al., 2012; Kelleher et al., 2013; although, see Sideli et al., 2012). McCarthy-Jones and Longden argue that the iron curtain between AVH in post-traumatic stress disorder (often termed “dissociative AVH”) and AVH in schizophrenia (so-called “psychotic AVH”) needs to be torn down, and that AVH should be included as a characteristic symptom of PTSD in diagnostic manuals. This is based on the argument that AVH in these two diagnoses often have a common phenomenology and a common root in traumatic life-events, which in turn leads to the argument that more trauma-based interventions should be made available to people diagnosed with schizophrenia distressed by hallucinations. Similarly, based on a review of the relationship between stressful life-events and the content of voices and visions, Steel argues that a subgroup of voices and visions, linked to stressful life-events, are likely to be responsive to trauma-informed interventions.

Cognitive behavioral therapy (CBT) remains the dominant psychological therapy for hallucinations. A number of papers in this Research Topic consider how CBT, which appears to only have a modest ability to help people with distressing hallucinations (Jauhar et al., 2014; van der Gaag et al., 2014), may be improved. Thomas, in a paper that has already stimulated significant interest, offers a thoughtful consideration of the problems of CBT for psychosis, examining when the CBT for psychosis protocol has uses and when it is limited. Fielding-Smith et al. explore how a better understanding of the role of the “self” (e.g., self-reflection, self-schema, self-concept) in causing and maintaining distress and disability in people with AVH may be able to improve the effectiveness of CBT. Smailes et al. examine how CBT for AVH may be tailored, based on the potential subtype of AVH that a person presents with, to improve its effectiveness.

As CBT interventions are increasingly being used in conjunction with mindfulness techniques (e.g., Shawyer et al., 2012), Strauss et al. examine the current evidence-base for mindfulness-based interventions for people distressed by hearing voices. The recent development of Relating Therapy for people with AVH (Hayward et al., 2009) is built upon by Hayward et al. who report on gender differences in how men and women relate to their voices (e.g., engagement vs. distancing) and consider the results of their findings for how psychological therapies could be tailored by gender. This work reflects an increasing interest in the relation between gender and hallucinations, psychosis more generally, and psychiatric diagnosis (Fisher et al., 2009; Marecek and Gavey, 2013; McCarthy-Jones et al.).

Finally, extending the collection beyond psychological therapies for hallucinations, a number of other therapeutic directions are considered. Koops et al. review the evidence base for transcranial direct current stimulation (tDCS) as a possible new treatment option to help reduce treatment-resistant auditory hallucinations. Taking a different approach, Waters et al. explore the potential for sleep interventions to improve the mental health of people with psychosis. There is evidence that almost all individuals with psychotic disorders meet the criteria for Insomnia Disorder and comorbid sleep complaints, and that sleep therapy can be effective in improving sleep, negative mood, and psychotic symptoms. Their paper explores the attitudes and preferences of people with psychosis toward three different types of treatment for sleep problems: (i) standard pharmacological interventions (antipsychotics, hypnotics, and sedatives), (ii) melatonin, and (iii) cognitive and behavior therapy (CBT). Waters et al. find that participants view both psychological and behavioral-type interventions and melatonin as valid adjuncts to pharmacotherapy and document a strong desire for participants to make their own choices regarding the type, and timing, of their therapies. Of course, in order for people to be able to choose between different therapies, these need to be made available. A right to choose in the absence of choices is worth little. This study also reinforces the point, made throughout this Research Topic, that people with hallucinations can be perceptive and insightful and must be consulted about their treatment and care.

In summary, the papers in this Research Topic offer a sample of the ways that the next generation of psychological therapies for hallucinations are developing. Specifically, they highlight how advances may stem from improved understandings of; (1) hallucinations themselves (as acts of communication, as having specific subtypes); (2) the person experiencing the hallucinations (their gender, multiple facets of their “self”); (3) inter-relations between the hallucinations and the experiencer (how the voices are related to, mindful responding), and; (4) the etiology of the hallucination (trauma-specialized interventions). Other promising forms of intervention include neurostimulation techniques, such as tDCS, and sleep-based interventions. When combined with other neurological and psychological developments in this field, which we would also refer the interested reader to, such as neurofeedback for hallucinations (McCarthy-Jones, 2012; Dyck et al.; Fovet et al.) and the Maastricht Interview (Romme and Escher, 2000), the future looks promising.

## Author contributions

All authors listed, have made substantial, direct and intellectual contribution to the work, and approved it for publication.

### Conflict of interest statement

The authors declare that the research was conducted in the absence of any commercial or financial relationships that could be construed as a potential conflict of interest.
